# A Survey on Digital Solutions for Health Services Management: Features and Use Cases from Brazilian National Literature

**DOI:** 10.3390/healthcare13182348

**Published:** 2025-09-18

**Authors:** Ericles Andrei Bellei, Cleide Fátima Moretto, Carla Maria Dal Sasso Freitas, Ana Carolina Bertoletti De Marchi

**Affiliations:** 1 Institute of Health, University of Passo Fundo (UPF), Passo Fundo 99052-900, RS, Brazil; 2 Institute of Informatics, Federal University of Rio Grande do Sul (UFRGS), Porto Alegre 90010-150, RS, Brazil

**Keywords:** health services administration, health information management, decision support systems, case managers, digital solutions, facilities and services

## Abstract

**Background and Objective:** Health services management faces increasing complexity, particularly in developing countries such as Brazil. Digital tools play a central role in optimizing health service operations, yet synthesized evidence on manager-focused applications remains limited. This study aimed to survey digital innovations for management within the Brazilian context. **Methods:** We systematically reviewed the complete proceedings of the Brazilian Symposium on Computing Applied to Health (SBCAS) from 2001 to 2024, identifying 26 studies that met eligibility criteria based on managerial relevance. **Results:** Applications identified predominantly addressed hospital management (e.g., resource scheduling and process optimization) and public health surveillance (e.g., disease prediction and monitoring), employing technologies such as machine learning and simulation. These tools primarily leveraged structured administrative data from national health information systems, reflecting existing data infrastructure capabilities. The reported implications suggest improvements in decision-making through optimized resource allocation (e.g., ICU beds and staffing), streamlined operational processes (e.g., bottleneck identification), enhanced planning and monitoring capabilities (e.g., endemic disease control and telemonitoring programs), and more timely, targeted public health surveillance (e.g., georeferenced analysis). **Conclusions:** The identified research aligns with global digital health trends but is also tailored to the complex realities of the healthcare system. Despite significant technical advancements, these digital solutions predominantly remain at the prototype stage, highlighting a gap between academic innovation and real-world deployment. Realizing the benefits of these tools will require a concerted effort to move beyond technical validation, focusing on implementation science, supportive policies, and strategic partnerships to integrate these solutions into managerial practice.

## 1. Introduction

The administration of modern healthcare services faces increasing complexity. Globally, systems are strained by rising patient volumes stemming from population expansion, demographic aging, and the increasing burden of non-communicable diseases, while concurrently navigating restrictive financial climates and regulatory mandates [1]. Within the context of developing nations such as Brazil, these pressures are compounded by endemic structural weaknesses [2], including insufficient funding mechanisms, significant geographical disparities in access and quality, and a mismatch between service demand and supply [3]. Particularly in resource-limited countries, the modernization and long-term viability of healthcare depend on harmonizing public investment with private sector participation, all underpinned by a robust framework [4]. This environment places responsibility on health service managers to balance clinical quality imperatives against financial performance targets, workforce limitations, and policy directives, often undertaking this critical task with limited analytical support [5].

Digital innovation offers powerful tools to address these multifaceted challenges, becoming integral to strategies aimed at optimizing health service operations. Purpose-designed digital platforms provide functionalities spanning the optimization of patient pathways, data-driven staffing models, automated supply logistics, financial analytics, and enterprise-level data integration [6]. Advanced artificial intelligence methods, such as machine learning and process discovery techniques, are poised to further elevate operational intelligence by converting diverse data sources into predictive insights. Yet, a notable asymmetry exists in the research landscape: despite a wealth of studies on clinical and patient-facing digital tools, the evidence base concerning technologies tailored for *managerial* decision-making is considerably less developed and remains fragmented [7,8]. This relative scarcity of empirical insight creates a blind spot for professionals guiding strategic planning and operational execution [9]. Also, recent literature highlights a research gap regarding digital health knowledge management for healthcare personnel [10].

Within Brazil, the *Brazilian Symposium on Computing Applied to Health* (SBCAS, in the Portuguese acronym) [11,12] has, since its inception, served as a premier forum for showcasing digital innovations tailored to the national health landscape. Its proceedings constitute a longitudinal record of technological solutions directed, yet no study has systematically synthesized that body of work at service management. There fore, this article closes introduces a survey on SBCAS archive to (I) catalog all contributions that address managerial challenges in health services, (II) map technical characteristics, implementation contexts, and reported outcomes, and (III) distill thematic trends and persisting evidence gaps. By organizing dispersed knowledge into a unified analysis, we aim to support decisions about future research and deployment strategies, seeking to contribute to optimizing decision-making processes [13], hampered by different limitations.

While previous studies have broadly examined digital health in Brazil, this study is the first to systematically synthesize digital tools developed specifically for health services management and administrative decision support. By focusing on the tools intended for managers rather than clinicians or patients, we fill a critical evidence gap and provide a unique perspective on the operational and strategic challenges being addressed by the national research community. The remainder of the paper is organized as follows. Section 2 details the search strategy, inclusion criteria, and analytical procedures. Section 3 reports the complete findings derived from 26 eligible studies, highlighting dominant technological domains, contextual drivers, and outcome patterns. Section 4 interprets these results in light of national and international digital health agendas, and Section 6 summarizes key contributions while outlining directions for subsequent research and practical implementation.

## 2. Methods

### 2.1. Study Design

This study used a scoping review methodology to systematically map and synthesize research on digital technologies applied to health service management in the Brazilian context. The review followed established guidelines for scoping reviews [14] to ensure transparency and rigor. As this study relied exclusively on publicly available published literature, institutional ethical approval was not required.

Building on a previous systematic investigation on digital health for healthcare management [15], guided by a registered protocol (OSF, https://osf.io/r8vc3, accessed on 17 August 2025), the present study adapts the established methodology. The prior protocol informed key aspects of this review’s design, including the refinement of the search strategy, the definition of eligibility criteria, and the data charting approach. This investigation, however, maintains a distinct focus, specifically analyzing the Brazilian context by examining contributions presented within the SBCAS proceedings. Such a focused approach is particularly relevant as the proceedings database, while a primary repository for Brazilian computing research, is not currently indexed in major international scientific databases (e.g., IEEE Xplore and the ACM Digital Library), potentially limiting the visibility of these national contributions in broader international reviews.

### 2.2. Database and Search Strategy

The primary information source for this review was the SBC Open Library, the digital repository for the Brazilian Computer Society (SBC). We encompassed all available SBCAS proceedings, a premier national forum for digital health innovations, from its inception in 2001 up to and including the year 2024. No language restrictions were initially applied; but, given the source, the included studies were primarily written in Portuguese or English.

The search was conducted within the SBC Open Library database, targeting the SBCAS proceedings. Due to search engine limitations requiring individual term searches, the following keywords were used sequentially, employing truncation syntax (*) to capture variations in both English and Portuguese: gest*, admin*, coord*, decis*, manager*, hospital*, depart*, institu*, center*, and public*.

### 2.3. Study Selection

The study selection process followed a systematic, multi-stage approach:

1. Identification and Duplicate Removal: The search string was executed on the SOL database; duplicate studies were identified and removed.
2. Title and Abstract Screening: Titles and abstracts identified through the search strategy were reviewed with a preliminary examination to determine whether they were related to the aim of this review.
3. Full-Text Assessment: Potentially relevant studies determined to be eligible based on the title or abstract were retrieved and evaluated with a full-text reading to settle their adequacy to the eligibility criteria.

### 2.4. Eligibility Criteria

Studies were included if they met the following criteria:

1. Target Audience: Includes features that primarily benefit health managers or policymakers as the intended users of the described technology.
2. Technological Focus: Centers around technology-based decision-support tools, software systems, analytical methods, or algorithms applied to healthcare management.
3. Application Context: Provides insights into real-world applications, specific use cases, implementation contexts, or deployments within health services.
4. Scope of Analysis: Offers information on the features, implementation challenges, or implications of the technologies within health systems management.

Studies were excluded based on the following criteria:

1. Patient or Clinical Focus: Primarily targets individual patients or clinical practitioners, rather than adopting a managerial, population, panel, or collective focus.
2. Lack of Real-World Application: Consists solely of theoretical proposals or descriptions without links to practical application or case studies.
3. Non-Technological Tools: Focuses exclusively on organizational policies, process redesign, or intervention programs that do not involve a specific technological tool or system as a core component.

### 2.5. Data Extraction and Synthesis

A structured data extraction form was developed using a spreadsheet and used to chart relevant information from the included studies:

- Article Metadata: The publication year, primary authors, and study location/context.
- Technological Characteristics: The specific type and name of the software, platform, algorithm, model, or method used or developed.
- Application Domain: The specific area within the health system (e.g., primary care, hospital administration, epidemiological surveillance, and outbreak prediction).
- Key Factors Considered: The main variables, indicators, criteria, or features central to the analysis, prediction, evaluation, or decision support.
- Implications for Practice: Reported or suggested impacts, recommendations, or potential benefits for health management practice, service delivery, or health policy.

Consistent with the objectives of a scoping review to map the existing literature, a formal critical appraisal of the methodological quality of included studies was not performed. However, the data extraction process captured information related to study design and validation approaches to provide context on the nature of the evidence. The extracted data were synthesized using a narrative approach combined with thematic analysis. Technological tools were categorized based on their primary function (e.g., data visualization, predictive modeling, and process optimization). Application domains were classified according to healthcare settings (e.g., primary care, hospital, and public health surveillance). Key factors, validation methods, and implications were grouped into recurring themes to identify dominant trends, common challenges, and evidence gaps related to managerial tools.

In addition to categorizing the extracted study characteristics, the underlying textual corpus was analyzed using network science methods to map conceptual relationships. The source text corpus was manually cleaned to remove non-pertinent sections, followed by technical pre-processing using regular expressions for n-gram reconstruction and Unicode normalization (NFC form). Co-occurrence data (collocates) were extracted using Voyant Tools with a 5-word context window and exported as a TSV file. These data were loaded using pandas, and lemmatization was performed on term and context columns using spaCy with the pt_core_news_md model. Lemma significance was calculated as the sum of contextual frequencies across all its appearances. The graph structure was derived by selecting the top 200 connections based on contextual frequency. An undirected, weighted network graph was constructed using NetworkX and spring_layout rendering in Matplotlib 3.10.6. We used lemmas as nodes and the selected co-occurrences as edges, applying a minimum degree of 1. Community structure was detected using the Louvain algorithm.

To explore the relationships between the key characteristics identified across the included studies, we performed a thematic co-occurrence analysis. We began by inductively coding the primary technology, application domain, and reported practical implications from each of the 26 studies summarized in the main table results, allowing studies to be assigned one or more categories within these dimensions (e.g., *prediction*, *hospital management*, and *resource optimization*). Based on this coding, we constructed a square co-occurrence matrix to generate a lower-triangle heatmap with Matplotlib 3.10.6 to quantify how frequently pairs of these derived categories appeared together within the same study.

## 3. Results

**Figure 1:**
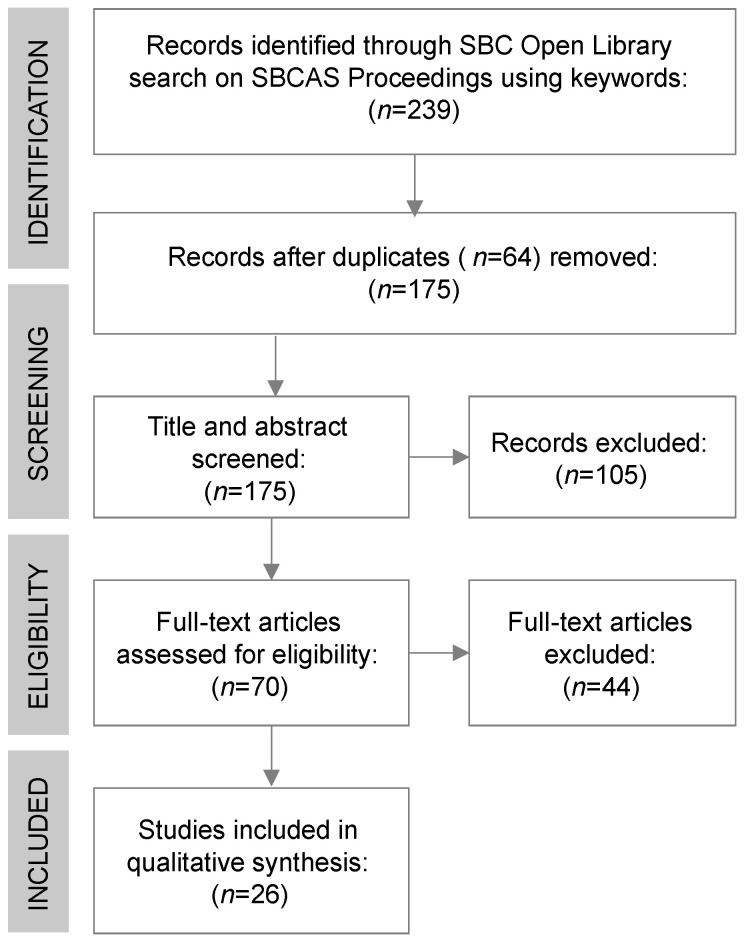
Flow diagram for the literature search and selection process.

The literature search yielded a total of 239 studies. At first, 64 duplicates were removed. Then, 105 studies were excluded during the review of titles and abstracts. Therefore, 70 full-text articles were assessed for eligibility. Of these, 44 studies were excluded due to any eligibility criteria, resulting in 26 studies included for synthesis. Figure 1 outlines the search and selection flow, while Table 1 summarizes the included studies [16,17,18,19,20,21,22,23,24,25,26,27,28,29,30,31,32,33,34,35,36,37,38,39,40,41].

**Figure 2:**
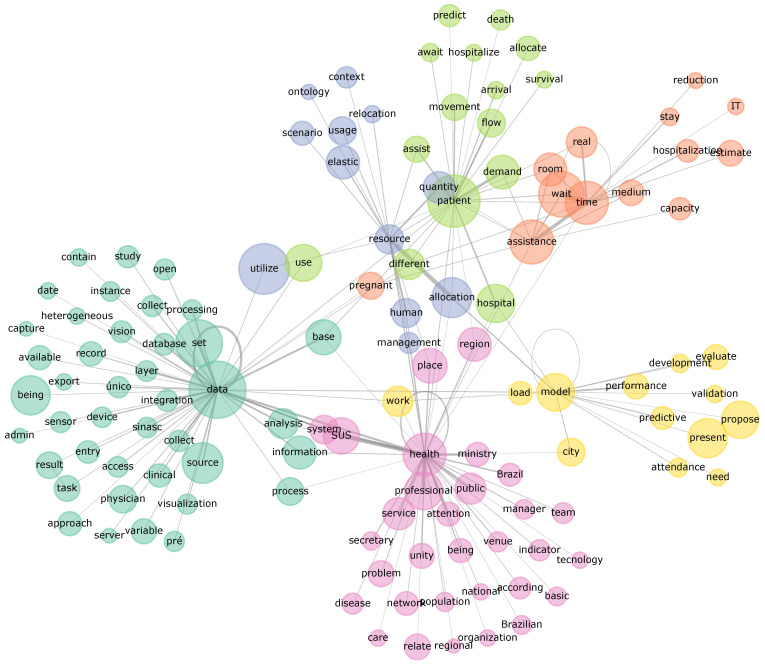
Network visualization of the top 200 lemmatized term co-occurrences translated into English. Node sizes correspond to lemma significance (summed contextual frequency), node colors indicate community membership (Louvain and Spring algorithm), and edge width reflects connection weight (contextual frequency). The vernacular version is available in the Supplementary Files.

Figure 2 illustrates the conceptual structure of the reviewed literature through a co-occurrence network of the 200 most significant terms. The analysis reveals several central hubs, with *health*, *data*, and *patient* emerging as the most prominent nodes, bridging various thematic clusters. The *health* node is tightly connected to terms defining the Brazilian context, such as *SUS*, *system*, *manager*, and *ministry*, anchoring the research in public health administration. Similarly, the *data* node connects to a large cluster of terms related to information processing, including *integration*, *database*, *source*, and *collect*. This structure highlights a clear pattern where technical data-handling methods are applied to patient-centered challenges within the overarching framework of the public health system.

Further analysis of Figure 2’s community structure, indicated by node colors, clarifies the primary research themes. The largest cluster (pink) represents the core domain of *public health services management*, linking administrative concepts (*manager* and *secretary*) with systemic elements (*system*, *SUS*, and *regional*). A second major cluster (green) focuses on *data and information infrastructure*, reflecting the technical processes required to utilize health records. A third distinct community (light green and orange) revolves around *hospital operations and resource logistics*, with terms like *patient*, *resource*, *allocation*, *demand*, *time*, and *capacity*. Finally, a fourth cluster (yellow) centers around *predictive modeling and validation*, evidenced by terms like *model*, *predictive*, *performance*, and *evaluate*. Together, these clusters show a research landscape focused on applying data processing and predictive analytics to optimize service delivery and resource management.

**Figure 3:**
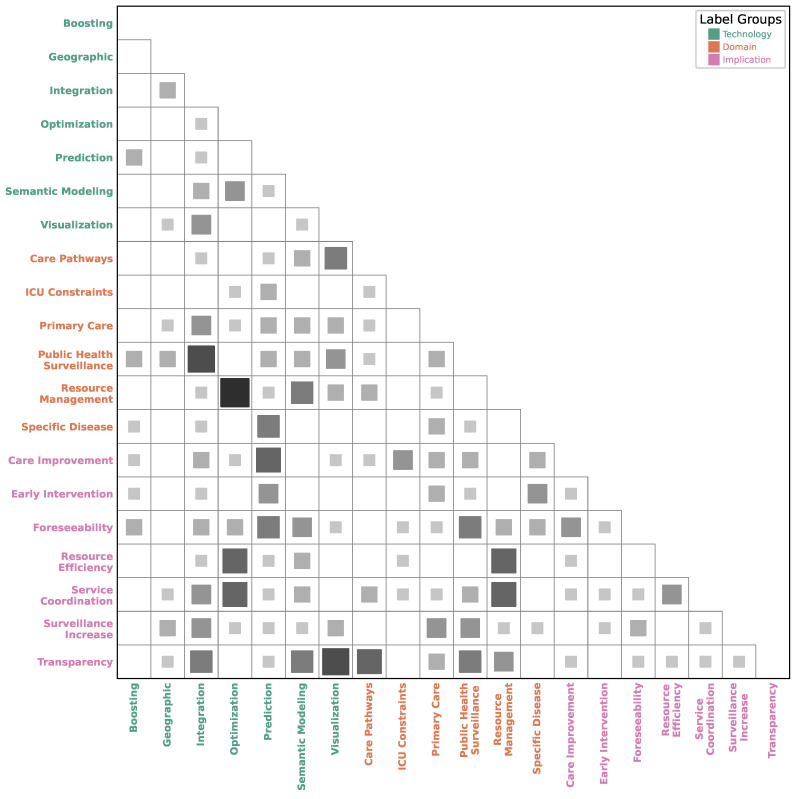
Lower-triangle visualization of the pairwise co-occurrence matrix between key characteristics classified from the studies. The color and area of each square are proportional to the absolute magnitude of the co-occurrence. Axis labels are grouped by color category.

Figure 3 presents a co-occurrence matrix that maps the relationships between the core characteristics of the reviewed studies, categorized as *technology*, *domain*, and *implication*. The visualization highlights dominant research paradigms by showing which concepts are frequently addressed together. One of the strongest associations observed is the triad of *optimization* technology, the *resource management* domain, and the implication of *resource efficiency*. This tight coupling indicates a significant focus on applying computational methods to improve the allocation and use of hospital resources. Similarly, *prediction* models are most frequently applied within the domains of *public health surveillance* and *specific diseases*, demonstrating a clear trend toward using machine learning for epidemiological forecasting and risk identification.

Further analysis of the matrix of Figure 3 reveals other important patterns. Technologies like *geographic* information systems and data *integration* techniques are strongly linked to the *public health surveillance* domain, underscoring the importance of spatial analysis and unified data for monitoring population health. The application of these technologies logically leads to implications such as *surveillance increase* and improved *service coordination*. Moreover, the figure shows a connection between the study of *care pathways* and *ICU constraints* and the use of *visualization* technology, suggesting an effort to map and understand complex patient flows. Overall, the heatmap illustrates a problem-driven research landscape where specific technologies are pragmatically applied to well-defined managerial domains to achieve targeted practical outcomes.

### 3.1. Application Domains

Analysis of the 26 included studies from the SBCAS proceedings reveals a landscape where digital health innovations are being developed to support the complex and dynamic realm of health service management within the Brazilian context. These studies predominantly leverage data from the Unified Health System (SUS), showcasing a reliance on diverse data types ranging from structured administrative records to semi-structured text reports. Common sources include national information systems such as the Hospital Information System (e.g., [32]), the Mortality Information System (e.g., [29]), the Information System on Live Births, the Notifiable Diseases Information System, the National Health Data Network, and Electronic Citizen Records (e.g., [38]), alongside outpatient production or dispensing records (e.g., [24,28]) and demographic census data (e.g., [29]). This reliance underscores the central role of established national and local databases in driving digital health research for management within Brazil.

Distinct patterns emerge, spanning a spectrum from core operational challenges to strategic public health, and targeting various managerial levels. Several studies focus on tools supporting strategic-level planning and policymaking, such as visualizing health regionalization [32] or integrating data for monitoring municipal indicators [29,38]. A significant portion of the research targets hospital management (10 studies), tackling challenges often relevant to both operational workforce and hospital administrators, including physician scheduling [34], inpatient accounting optimization [21], ICU bed allocation [40], inpatient flow prediction [41], dynamic resource allocation [26,36,39], optimizing surgical center capacity [18], and network traffic management [20].

Public health surveillance and disease control constitutes another major focus, with tools often supporting epidemiologists, program managers, and public health officials through georeferenced analysis [17], predictive modeling [33,37], COVID-19 data analysis and risk prediction [29,30], endemic control systems used by managers and endemic field agents [35], and integrated telemonitoring platforms [38]. Primary care applications included geographic systems-based territorial mapping [16], simulation for indicator balancing [23], and predictive models often providing clinical-level support alongside managerial insights, such as predicting gestational diabetes or neonatal mortality [28,31]. Similarly, tools for predicting ICU mortality risk [22] or congenital syphilis risk [37] primarily offer clinical decision support but have implications for resource management. Foundational work on data integration and knowledge management [24,25] and analyses of healthcare networks, patient trajectories, or regionalization [19,27,32] provide broader support across different managerial functions.

### 3.2. Technological Approaches

To address these managerial challenges, a range of technological approaches was observed, utilizing the varied data characteristics available. Using AI (artificial intelligence) and machine learning represents a prominent technological trend [22,28,30,31,33,37,41] predominantly applied to predictive tasks offering foresight into future events. These models often processed diverse inputs, combining individual-level clinical and sociodemographic data (e.g., from specific programs [37] or ICU databases [22]), historical case data, aggregated epidemiological indicators (morbidity and mortality), resource usage metrics (patient flow and room usage), and even climatic factors. Models like LightGBM, XGBoost, random forests, and CNNs were frequently employed. Simulation, particularly discrete-event simulation, often combined with BPMN, was used for process analysis and resource optimization [18,21,23], typically modeling process activities, time consumption, and resource utilization derived from structured operational data. Geographic information systems facilitated spatial analysis for primary care and epidemiology [16,17], integrating aggregated health indicators or case counts with territorial boundaries. Some geographic applications also incorporated semi-structured data alongside structured information [17]. Semantic technologies (ontologies and knowledge graphs) addressed SUS data integration challenges [25,29], aiming to create unified views from disparate, often structured, sources like mortality and notification data [29]. Other identified technologies included custom web platforms [32], mobile applications [35], constraint programming [34], complex network analysis [19], and software-defined networking [20].

Predictive models were often presented as tools to assist clinical and managerial decision-making by anticipating risks, such as mortality in ICUs or during the COVID-19 pandemic [22,30], predicting disease outbreaks or congenital syphilis cases to guide interventions [33,37], forecasting inpatient admissions to streamline administrative processes [41], or enabling early identification and monitoring of at-risk patients in primary care [28,31]. Furthermore, foundational tools aimed to improve data integration, knowledge management, and system understanding, facilitating transparent analysis for health technology assessment [24], enabling flexible integration of diverse SUS data sources [25], supporting evidence-based policy in health regionalization [32], or providing a better understanding of patient care trajectories and network dynamics [19,27].

### 3.3. Maturity and Implications

The maturity and validation of these diverse technological approaches also varied across the included studies, reflecting different research stages and priorities. Quantitative performance metrics (e.g., accuracy and sensitivity) were standard for evaluating machine learning models [22,28,30,31,33,37,41]. Simulation or modeling studies typically demonstrated value through scenario analysis or computational simulation [18,21,23,40]. Several projects relied on case studies with expert or manager feedback [17,29,38] or presented descriptive analyses, system screens, or visualizations [16,32,35], indicating a focus on feasibility. This heterogeneity ranges from technically validated algorithms suggesting higher technical maturity to system prototypes assessed primarily through qualitative feedback or demonstration, likely indicating earlier development stages.

The reported implications converge on enhancing service management through various mechanisms. Several studies highlighted the potential for improved resource allocation and operational efficiency, whether through optimized physician scheduling [34], better bed allocation [40], dynamic staff deployment based on predicted demand [26,39], identification of bottlenecks in administrative processes like hospital billing [21], or improved network performance for critical medical equipment [20]. Others emphasized enhanced planning and monitoring capabilities, such as improved epidemiological surveillance through georeferenced tools [17], better balancing of primary care indicators using simulation [23], optimized endemic control actions via mobile reporting systems [35], or enabling remote monitoring of health programs [38].

## 4. Discussion

The results revealed a diverse landscape of digital solutions, with both converging themes and disparities across studies. In this discussion, we elaborate on the strengths and limitations observed in each thematic cluster of solutions, compare these findings with existing literature trends, and highlight gaps in evidence that warrant attention. Therefore, we aim to synthesize how these SBCAS studies collectively advance the agenda of health services management. As some categories have a limited number of representative studies, caution is needed when interpreting the findings, as they are more exploratory in nature.

### 4.1. AI and Machine Learning Applications

A prominent theme is the use of AI and machine learning to inform managerial decisions. Numerous included studies applied models for predictive analytics, for example, forecasting neonatal mortality or hospital admissions, using datasets from public health information systems. These efforts mirror a global trend of leveraging AI for healthcare operations: predictive algorithms have been shown to improve scheduling efficiency and reduce costs in hospitals [42,43]. In the review, the AI models (ranging from tree-based ensembles to deep neural networks) often outperformed traditional heuristics or risk scores, suggesting a strength in their potential to provide foresight (e.g., early warnings of ICU bed demand or disease outbreaks). However, their limitations are equally evident. Most were developed and tested on retrospective data in silos, with few reports of integration into live workflows or real-time decision support. This gap between model development and deployment reflects a broader challenge: globally, AI-based decision support has made deeper inroads in clinical care than in administrative arenas [44]. While clinical decision-support systems are increasingly common at the point of care, analogous tools for managerial decision-making remain in nascent stages [45]. Brazilian studies attempt to bridge this asymmetry by applying AI to managerial problems, aligning with international calls to extend data-driven support to operational planning [46]. Still, moving from promising pilot results to sustained use will require robust validation, user trust, and seamless integration with hospital information systems.

### 4.2. Optimizing Hospital Operations

In total, 10 of the 26 studies targeted hospital operations, tackling scheduling, resource allocation, patient flow, and related logistical challenges. They employed techniques like discrete-event simulation for process optimization, mathematical modeling for staff rostering, and even network engineering (software-defined networking) to prioritize critical device traffic. A common strength is the quantitative insight these tools provide, for instance, process simulations highlighted bottlenecks in hospital workflows, and algorithms demonstrated measurable reductions in wait times. Such findings are consistent with international literature where simulation and heuristics have improved operational metrics in controlled studies [47].

The SBCAS contributions confirm that even in middle-income settings, modeling patient pathways or optimizing bed management can identify efficiency gains similar to those reported in high-income hospitals. A distinguishing aspect of the SBCAS studies is their focus on local context constraints, for example, tailoring surgical scheduling to regional hospital capacities or modeling ICU bed allocation under SUS regulations. This context-specificity is valuable, yet it also underscores a limitation: solutions are often bespoke and validated only in a few institutions. By contrast, nearly one-fifth of US hospitals had adopted some form of AI by 2022 [48]. In this matter, data interoperability issues within hospital systems (discussed further below) could hinder the integration of these optimization tools into electronic health records or hospital management systems.

### 4.3. Public Health Surveillance and Primary Care

Another cluster of studies concentrated on public health and primary care management. These included geospatial dashboards for epidemiological surveillance, predictive models for community health risks (such as gestational diabetes incidence), and decision-support tools for primary care planning (such as balancing preventive care targets). The strength of this line of work lies in its alignment with pressing public health needs, where digital platforms can enhance disease surveillance. As the example of the SARS-CoV-2 pandemic demonstrates, timely data dashboards and risk prediction systems can inform policy responses [49]. The reviewed studies demonstrate that SBCAS researchers utilize digital maps and risk algorithms to assist public health officials in identifying hotspots and anticipating service demand. For instance, one study [38] integrated notification and mortality data to improve epidemiological monitoring of endemic diseases at the municipal level, potentially informing resource allocation more proactively. Internationally, such approaches resonate with the increasing use of spatial analysis and AI in public health, from early outbreak detection to allocating community health workers based on predictive risk stratification [50,51]. A key similarity is the recognition that primary care and preventive services benefit from data-driven targeting of interventions [52]. This managerial focus speaks to Brazil’s efforts to equip health service administrators with actionable intelligence.

### 4.4. Strategic Planning and Resource Allocation

The drive to improve efficiency and resource use is not unique to Brazil; it is seen worldwide as health systems seek to do “more with less” amidst growing demands. For instance, the WHO’s digital health strategy 2020–2025 advocates for systems that enhance health system management and policy [46] (p. 27). From the findings, in addition to operational tools, several studies addressed higher-level strategic management needs. Examples include systems for visualizing health service regionalization, tools to help balance healthcare indicators across regions, and platforms to support policy-making (such as planning primary care coverage or monitoring municipal health performance against targets).

These strategic tools are particularly important in a country as large and decentralized as Brazil, where planners must allocate limited resources across diverse regions. Notwithstanding, this is a challenge observed in other countries, such as Italy, which also faces a fragmented nature of the hospital facilities, highlighting infrastructural and managerial differences [53]. The reviewed studies show an encouraging focus on this macro-level decision support. One study [32], for instance, developed a web-based interface to explore inter-regional patient flow and service availability, aligning with Brazil’s regionalization policies. Another one [23] used simulation to automatically rebalance primary care targets, an approach that could guide policymakers in setting realistic goals for clinics. The strength of these initiatives lies in their system-wide perspective. They attempt to give health system managers (at municipal, state, or federal levels) the analytical tools that corporations often use for strategic planning. Internationally, there are parallels in the form of health system dashboards and planning models. Many countries use aggregated data systems (like the WHO’s Health Observatories or the DHIS2 platform in dozens of low- and middle-income countries) to inform strategic decisions and monitor performance indicators [54]. The Brazilian studies are consistent with this global trend, aiming to transform data into actionable insights for health administrators. A difference, however, is that there is little evidence that these tools have been officially adopted in larger scenarios. Nonetheless, the studies reviewed provide a foundation that future work can build on.

### 4.5. Data Integration and Semantic Technologies

Fragmentation of health data systems is a recurring barrier highlighted both in our review and in international literature [55]. Several Brazilian studies directly tackled this issue by developing data integration solutions, including the use of ontologies and knowledge graphs to unify disparate databases. These semantic approaches aimed to bridge information from the national health system (SUS), linking primary care records, hospital admissions, and disease notification datasets into a cohesive knowledge base. The strength of this strategy is its ambition to achieve technical interoperability in a context where proprietary standards have historically been absent [56]. By creating a semantic layer, the studies sought to enable cross-system queries for managers, mirroring global efforts to use standardized data models for better interoperability [57].

However, the reviewed studies, which focus on technical proofs-of-concept, do not fully capture the primary obstacles to interoperability at scale. The most significant barriers are not technical but are rooted in governance, policy, and sustainable financing [58]. While Brazil’s National Digital Health Strategy and the creation of the National Health Data Network represent critical top-down policy efforts to standardize data exchange, their effective implementation is often hindered by a lack of clear governance models for data stewardship and inconsistent enforcement [58]. Furthermore, at the municipal and state levels, chronic underfunding for IT infrastructure means that health facilities are often unable to upgrade the legacy systems or invest in the workforce training necessary to comply with new national standards [59]. Without dedicated financing to support this transition, even the best-designed semantic solutions remain impractical for many health service managers [59].

Globally, interoperability remains an unsolved challenge, confirming that these issues are not unique to Brazil. Even in high-income countries with strong policy mandates, health data exchange is often incomplete and labor-intensive, as documented in an Ohio-based study where stakeholders reported resorting to manual portal use due to a lack of EHR integration [60]. Brazil’s semantic interoperability experiments are innovative but their real-world impact is contingent on the maturation of the RNDS and broader SUS reforms that address these systemic barriers. The WHO’s push for standardized health information architecture provides a useful framework [46] (p. 21); but, as international trends suggest, its success depends on strong political will and a robust financial commitment. Absent such alignment, the knowledge graphs developed in academic settings risk remaining isolated exercises rather than becoming foundational components of an integrated national health system.

### 4.6. Maturity, Readiness, and Barriers to Real-World Implementation

Related to the issue of implementation is the overall maturity level of the surveyed solutions. Most fall into early-stage development, a status evidenced by the scarcity of reports on real-world performance or scalability. This prototype paralysis is not merely an academic artifact, but a symptom of significant real-world adoption barriers [61], such as insufficient funding, fragmented IT infrastructure, and resistant organizational cultures, which were outside the scope of the analyzed studies [62]. From an implementation science perspective, the journey from a validated algorithm to a trusted managerial tool requires overcoming challenges of user acceptance, providing adequate training, and ensuring organizational readiness, factors that determine whether a digital solution is ultimately adopted or abandoned [63]. Despite the inability to empirically assess the technologies described in the studies, the experimental descriptions suggest that the created tools do not surpass the level four or five threshold of the technology readiness level (TRL) methodology. Ranging from one to nine, these levels typically indicate technologies that were validated in a lab or relevant environments, but they are still distant from achieving the highest level, where the technology is proven in an operational environment [64].

In the international literature, there is growing concern about the lack of longitudinal evidence and real-world evaluation for digital health tools [65]. The successful implementation of the DHIS2 in dozens of low- and middle-income countries, for instance, was not solely a technical achievement. Its success was underpinned by strong governance, sustained training programs, and a focus on stakeholder needs at all levels, which fostered buy-in and ensured the platform’s utility in diverse settings [54]. Similarly, the push for adopting interoperability standards such as HL7-FHIR in Europe and North America highlights that technical specifications alone are insufficient. Widespread adoption depends on national policy mandates, ecosystem-wide coordination, and addressing complex sociotechnical factors that often prove more challenging than the technology itself [60]. For BRICS nations, which include Brazil, the literature recommends that policymakers support the development of technologies such as AI, which may lead to significant changes in management and production practices [66].

These international cases suggest that for the innovations identified in our review to mature, a more holistic strategy is required for Brazil. It involves moving beyond technical validation to co-designing solutions with end-users, securing long-term funding, and fostering a data-driven culture within health institutions. Several of the reviewed Brazilian studies implicitly acknowledge these hurdles by including, for example, user feedback stages or pilot usability tests, but none provided a thorough evaluation of user satisfaction or training needs. International experience shows that these factors often determine the success or failure of digital health initiatives. For instance, introducing a clinical decision support system required multidimensional change management, including engaging frontline providers and aligning the tool with existing processes [67]. To progress further, future projects must test tools in real operational environments, measure key performance indicators over time, and refine solutions based on continuous feedback. Engaging stakeholders (from IT departments to health managers) early and throughout deployment is essential for translating promising prototypes into dependable and scalable decision-support tools that are truly integrated into everyday health service management.

## 5. Limitations

This study has several limitations that should be considered when interpreting its findings. The primary limitation is the exclusive focus on the proceedings of the SBCAS. This choice was deliberate, intended to capture a rich, context-specific body of work from a premier national forum known for its authority within the Brazilian Computer Society and its specific relevance to health informatics. However, this focus inherently narrows the scope of the review. Consequently, the findings may not be fully representative of all digital health research conducted in Brazil. The conclusions should be understood as a profile of the research priorities and technological trends driven within the SBCAS community, rather than an exhaustive overview of the entire national landscape, as it is plausible that research in certain domains might also be published in other venues. Furthermore, while our review covers an extensive period, it provides limited longitudinal evidence regarding the real-world adoption and impact of the reported solutions. To build on this work, future research should adopt a broader scope by expanding the search to include other relevant databases.

## 6. Conclusions

This study synthesized digital solutions developed for health services management in Brazil from the SBCAS literature, highlighting diverse approaches from artificial intelligence and machine learning to semantic data integration and operational optimization. Collectively, the studies illustrate significant technical advancement, particularly regarding predictive analytics, hospital resource management, public health surveillance, and strategic planning tools. The thematic directions seen in SBCAS research are aligned with global trends, particularly in striving for enhanced decision support, improved data integration, and a shift of digital innovation toward health services management. This strategic shift is especially critical within the Brazilian SUS, a system of continental dimensions whose principles of universality and decentralization create immense managerial complexity. In this context, data-driven tools are not merely optimizations but are fundamental for addressing persistent challenges of resource allocation, overcoming vast regional inequalities, and ensuring the long-term sustainability of public healthcare delivery.

The SBCAS community has demonstrated alignment with international digital health priorities, addressing critical managerial domains. A clear emphasis has emerged on creating solutions tailored explicitly to the local realities and complexities of the SUS, including context-specific simulations and AI-driven forecasting systems. This contextualization represents a critical strength, as it positions local innovations to address actual needs and constraints faced by health administrators. To translate this potential into practice, future research should move beyond prototype development and toward implementation science, forging stronger partnerships with health authorities to co-design and test tools that integrate seamlessly into existing managerial workflows.

However, several gaps remain. The studies collectively demonstrated limited progress toward real-world implementation and evaluation. Many proposed digital solutions have remained at experimental stages, lacking longitudinal follow-up or practical considerations for scalability. Closing this implementation gap requires more than just refined research methodologies; it calls for a clear strategy to guide digital adoption in management, ensuring that policy and funding can support the transition from promising research to scalable, system-wide solutions. Similarly, sociotechnical dimensions, such as user acceptance and organizational readiness, remain underexplored. Future research must address these limitations by incorporating longitudinal evaluations and user-centric methodologies to ensure that technological advancements deliver tangible benefits to health service management in Brazil.

## Figures and Tables

**Table 1:** Summary of the included studies that presented digital solutions for health services management in Brazil.

Author	Year	Tool, Technology, or Technique	Application Domain & Scope	Key Factors or Variables Used	Foundation or Validation Technique	Implications for Practice
Carvalho et al. [16]	2015	Territorial digital map of family health strategy; geographic information systems	Primary health care, leprosy control; Castanhal, PA, Brazil	FHS territories, health data, risk factors	GIS-based visualization and analysis	Enhanced information management for health programs, leprosy control.
Inácio et al. [17]	2016	GISTelemed (georeferenced epidemiological analysis tool)	Telemedicine, epidemiological view; Santa Catarina state	Structured and semi-structured data, morbidity, mortality	Case study or feedback from experts	Improved epidemiological surveillance and resource allocation.
Redeker et al. [18]	2017	Business process modeling and simulation; BPMN, discrete-event simulation	Healthcare capacity and resource allocation	Surgical center capacity, performance indices	Simulation of ”what if” scenarios	Optimized resource use and improved healthcare process management.
Jasmim et al. [19]	2017	Analysis of healthcare network; complex network analysis	Healthcare network characterization	Patient flow, service types, professionals, patients	Exploratory study of patient flows	Better understanding of healthcare network behavior and resource allocation.
Oliveira et al. [20]	2018	Software-defined networking (SDN)	Hospital network management, prioritization of traffic	Medical device traffic, network quality parameters	SDN-based traffic prioritization	Improved network quality of service for critical devices.
Vargas et al. [21]	2018	BPMN, discrete-event simulation	Analysis of billing process, hospital accounting	Process activities, time, resource usage	Proof simulations	Identification of bottlenecks and improvement in billing process.
Schmidt et al. [22]	2018	Deep learning model (CNN)	Mortality prediction in intensive care units; MIMIC III database	Patient data, risk of death	Comparison with APACHE II model	Improved prediction of mortality risk in ICUs.
Veras et al. [23]	2019	Tool for automatic goal balancing; Simulation of contingent scenarios	Health indices in primary care	Health indicators, planned values, deviations	Statistical significance tests	Improved planning and monitoring of indicators in dynamic scenarios.
Ferré et al. [24]	2020	Platform for knowledge management and visualization; data extraction, wiki documentation	Health technology assessment, SUS data; Brazil	Outpatient records, clinical protocols, dispensing records, user data	Automated extraction and visualization, documented in wiki	Transparent knowledge management and reproducible statistical analysis for decision-making.
Rolim et al. [25]	2020	Enterprise knowledge graph; incremental construction, ontologies, semantic integration	SUS data integration; Brazil	Data from SIM and SINASC, health data sources	SPARQL queries for validation	Flexible and extensible approach for integrating various SUS data sources.
Fischer et al. [26]	2020	Helastic model; IoT and elasticity in cloud computing	Human resource analysis in intelligent hospitals	Patient room usage, healthcare professional availability	Predictive approach for resource movement	Reduced waiting times and optimized resource allocation.
Silva et al. [27]	2020	Analysis of pregnant women’s care trajectories; data analysis	Healthcare network analysis for pregnant women; São Paulo	Patient trajectories, attendance records, health units	Analysis of patient trajectories	Better visibility of the healthcare system.
Moreira et al. [28]	2021	Machine learning models for type 1 diabetes prediction; classification model	Diabetes prediction in pregnancy	Outpatient production data, patient characteristics	Sensitivity, and precision of the classifier	Early prediction and better care to pregnant women at risk.
Gomes et al. [29]	2022	ONTOVID (semantic knowledge graphs); NeOn methodology, ontology-based data integration	COVID-19 data analysis, mortality information	Mortality data, COVID-19 notifications, vaccinations, hospitalizations	Validation by health department managers	Improved data integration and accurate extraction of indicators.
Rodrigues and Kreutz [30]	2022	Predictor for mortality risk in COVID-19; random forests	COVID-19 mortality risk classification; Brazil	Patient data, risk of death	AUC-ROC score for model evaluation	Assist in decision-making in the hospital environment.
Moreira et al. [31]	2022	Prediction of neonatal death; classifier using SUS and census data	Neonatal death prediction	SUS information systems data, demographic census data	Accuracy and sensitivity of prediction	Early warning system for neonatal risks and improved maternal/newborn monitoring.
Pereira et al. [32]	2023	Web platform for visualization and analysis; complex system techniques	Health regionalization; Brazil	Patient flow records from SIHSUS, health regions	Visualization and interactive web platform	Support evidence-based public policymaking in health regionalization.
Aleixo et al. [33]	2023	Machine learning models (LightGBM, XGBoost, Catboost); statistical analysis	Dengue outbreak prediction, congenital syphilis diagnosis	Sociodemographic, climatic, historical case data, mosquito index	AUC metric for syphilis, outbreak identification accuracy for dengue	Improved prediction of disease outbreaks and diagnosis of congenital syphilis.
Cid et al. [34]	2023	Constraint programming the in physician rostering problem	Physician scheduling in hospitals	Service demand, physician availability, scheduling preferences	Test and proof	Improved efficiency and fairness in physician scheduling.
Gregório et al. [35]	2023	Information system for endemic control; app for health agents	Aedes aegypti control; Itajubá, MG, Brazil	Epidemiological data, weekly reports, field data	Data collection and reporting system	Enhanced monitoring of endemic control actions.
Fischer et al. [36]	2023	CityHealth model; elasticity concept in cloud computing	Hospital management in smart cities	Vital signs data, hospital resource usage, patient demand	Emulation of smart city with hospitals	Reduced waiting times and improved resource management.
Teixeira and Endo [37]	2023	Machine learning models (SVM, AdaBoost); Statistical analysis	Prediction of congenital syphilis; Pernambuco, Brazil	Clinical and sociodemographic data of pregnant women	Performance evaluation of different models	Assist in resource allocation and optimize healthcare actions in low-resource settings.
Alencar et al. [38]	2024	Telemonitoring system; data extraction from RNDS, PEC, SINAN	Telemonitoring of health programs; Manaus, AM, Brazil	Patient data, reports, graphs, municipal indicators	Real-time reporting and graphing	Remote monitoring of patients and support for decision making by health managers.
Fischer et al. [39]	2024	ElCareCity model; Reactive and proactive elasticity	Multi-hospital resource management in smart cities	Patient usage of health environments, personnel allocation	Emulation of smart city with hospital environments	Efficient allocation of health professionals and reduced waiting times.
Gomes et al. [40]	2024	Mathematical modeling for ICU bed allocation optimization	ICU bed allocation	Bed types, occupancy rate, patient survival rates	Computational simulation with real data	Improved ICU bed allocation and patient survival rates.
Consoli et al. [41]	2024	Prediction of inpatient admissions; AI solution	Inpatient flow prediction	Patient data, length of stay	Accuracy of prediction	Accelerated administrative processes and improved care.

## Data Availability

Preliminary and exploratory results of this study were presented at the 2025 edition of the SBCAS conference in the form of a short ongoing paper. DOI 10.5753/sbcas.2025.7726.

## Supplementary Materials

The following supporting information can be downloaded: https://www.mdpi.com/article/10.3390/healthcare13182348/s1: Figure S1: Network visualization in vernacular language (Brazilian Portuguese); file S1: occurrence codes used to generate Figure 3.

## Abbreviations

The following abbreviations are used in this manuscript:

AI	Artificial Intelligence
BPMN	Business Process Model and Notation
DHIS2	District Health Information System 2
CNN	Convolutional neural network
FHS	Family Health Strategy (primary care program)
HL7-FHIR	HL7 Fast Healthcare Interoperability Resources
ICU	Intensive Care Unit
OSF	Open Science Framework
SBC	Brazilian Computer Society
SBCAS	Brazilian Symposium on Computing Applied to Health
SUS	Unified Health System (of Brazil)
TRL	Technology Readiness Level
WHO	World Health Organization
