# Supplementary figures and images for: A Survey on Digital Solutions for Health Services Management: Features and Use Cases from Brazilian National Literature
Source: Healthcare (Basel). 2025 Sep 18;13(18):2348. doi: 10.3390/healthcare13182348 (PMC12469461; doi:10.3390/healthcare13182348)

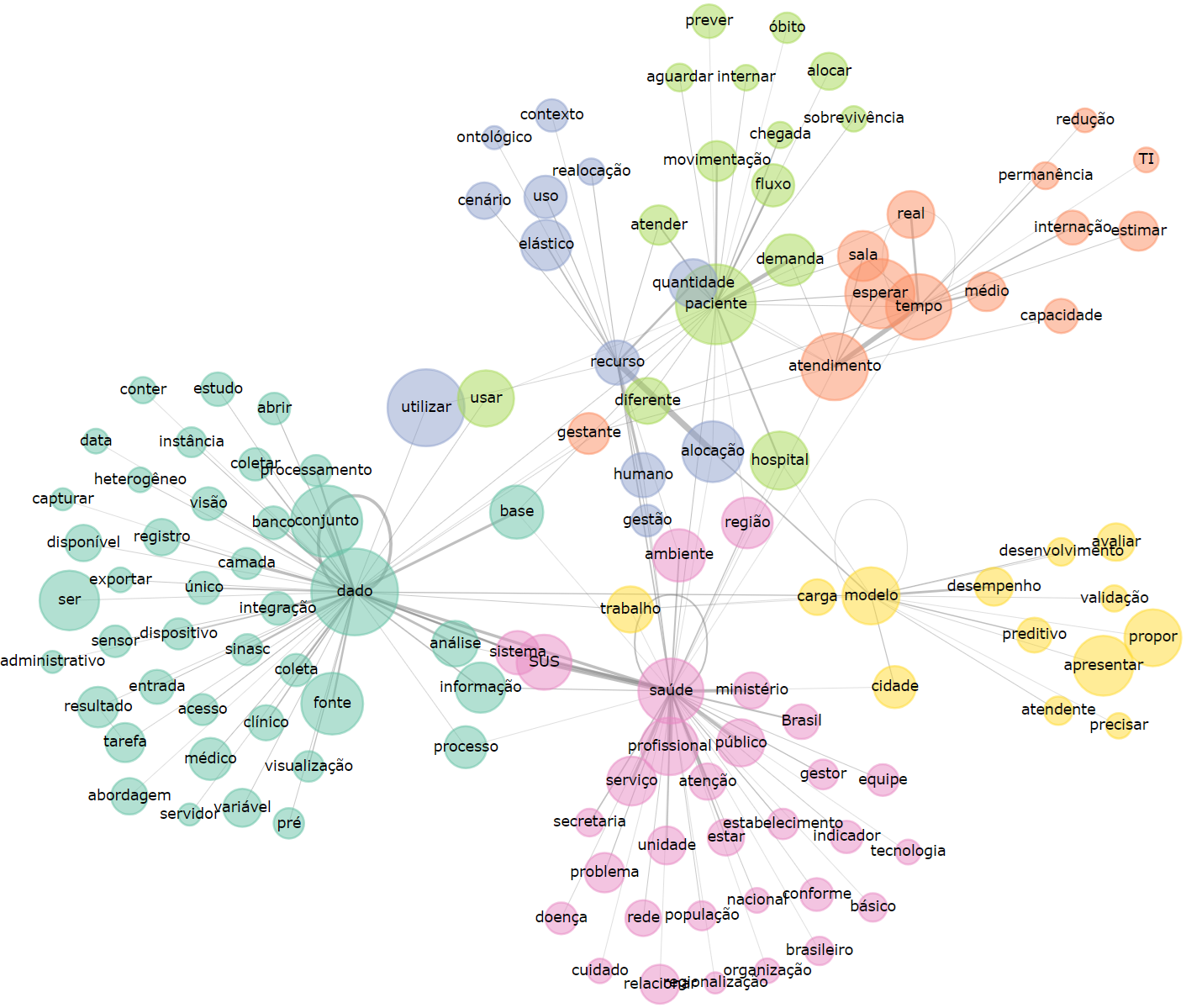

Supplement: Supplementary file 1 [file healthcare-13-02348-s001.zip › healthcare-3792259-supplementary.png]
